# Supplementary material for: Ammonia and Particulate Matter Emissions at a Korean Commercial Pig Farm and Influencing Factors
Source: Animals (Basel). 2023 Oct 27;13(21):3347. doi: 10.3390/ani13213347 (PMC10649674; doi:10.3390/ani13213347)
Supplement: Supplementary file 1 [file animals-13-03347-s001.zip › animals-2669095-SI.pdf]

animals

Article

Ammonia and Particulate Matter Emissions at a Korean Commercial Pig Farm and Influencing Factors

Lak-yeong Choi [1,2,†], Se-yeon Lee [1,2,†], Hanna Jeong [1,2], Jinseon Park [2,3], Se-woon Hong [1,2,3,*], Kyeong-Seok Kwon [4], and Mijung Song [5]

[1] Department of Rural and Bio-Systems Engineering, Chonnam National University, Gwangju 61186, Republic of Korea; cly6847@jnu.ac.kr (L.-y.C.); seyeonn@jnu.ac.kr (S.-y.L.); 208769@jnu.ac.kr (H.J.); hsewoon@jnu.ac.kr (S.-w.H.)
[2] Education and Research Unit for Climate-Smart Reclaimed-Tideland Agriculture, Chonnam National University, Gwangju 61186, Republic of Korea; icarus381@jnu.ac.kr
[3] AgriBio Institute of Climate Change Management, Chonnam National University, Gwangju 61186, Republic of Korea
[4] Animal Environment Division, National Institute of Animal Science, Wanju, 55365, Republic of Korea; kskwon0512@korea.kr
[5] Department of Environment and Energy & Department of Earth and Environmental Sciences, Jeonbuk National University, Jeonju 54896, Republic of Korea; mijung.song@jbnu.ac.kr
* Correspondence: hsewoon@jnu.ac.kr; Tel.: +82-62-530-2183; Fax: +82-62-530-2159
† These authors contributed equally to this work.

Supplementary Materials

Table S1. All measurement data obtained from piglets. "n/a" indicates cases where there are no measurement results or they were lost due to errors in the measurement process. (NA: number of animals, AA: age of animals in days, VR: ventilation rate in $m^3 h^{-1}$, OT: outdoor air temperature in °C, OH: outdoor relative humidity in %, IT: indoor air temperature in °C, IH: indoor relative humidity in %, OC: outdoor concentration in ppm for ammonia and μg m^{-3} for PMs, EXC: concentration at air outlets in ppm for ammonia and μg m^{-3} for PMs, EC: emission concentration in ppm for ammonia and μg m^{-3} for PMs, and EF: emission factor in kg $animal^{-1}$ yr^{-1}).

Date (yy.mm.dd)	NA	AA	VR	OT	OH	IT	IH	OC				EXC				NH_3		TSP		PM_{10}		$PM_{2.5}$	
								NH_3	TSP	PM_{10}	$PM_{2.5}$	NH_3	TSP	PM_{10}	$PM_{2.5}$	EC	EF	EC	EF	EC	EF	EC	EF
20.05.21	313	23	3394	23.6	54.5	27.9	60.0	0.00	35.03	20.63	10.88	13.87	322.72	184.27	134.68	13.87	0.91	287.69	0.03	163.65	0.02	123.81	0.01
20.06.05	421	33	16617	29.2	57.4	31.2	55.4	0.36	196.22	127.00	27.60	1.76	291.28	n/a	105.61	1.40	0.33	95.06	0.03	n/a	n/a	78.01	0.03
20.06.16	421	44	11601	27.3	52.7	29.1	60.9	0.12	80.02	37.63	18.38	2.31	253.63	70.02	51.65	2.19	0.36	173.61	0.04	32.39	0.01	33.28	0.01
20.06.23	421	51	16617	30.7	50.3	30.0	55.1	0.22	102.02	30.13	16.63	1.54	246.97	100.69	n/a	1.32	0.31	144.95	0.05	70.56	0.02	n/a	n/a
20.08.20	383	47	18728	34.8	56.6	32.7	66.6	0.15	58.22	23.38	16.00	1.41	144.16	87.77	58.87	1.26	0.36	85.94	0.04	64.40	0.03	42.87	0.02
20.08.28	383	55	17582	32.8	67.2	32.1	71.7	0.00	35.96	17.91	7.73	1.33	72.76	53.78	22.84	1.33	0.36	36.79	0.01	35.87	0.01	15.12	0.01
20.09.24	403	26	4399	26.0	56.6	27.6	70.4	0.01	42.93	15.00	4.67	10.81	145.39	71.49	31.14	10.80	0.71	102.46	0.01	56.49	0.01	26.47	0.00
20.10.06	404	38	2950	19.7	46.0	29.6	76.0	0.00	38.49	30.36	24.40	8.81	968.38	377.48	86.04	8.81	0.40	929.89	0.06	347.13	0.02	61.64	0.00
20.10.22	389	54	2771	17.5	64.7	28.1	77.5	0.00	188.17	124.00	34.86	7.56	645.88	436.12	121.54	7.56	0.33	457.71	0.03	312.12	0.02	86.68	0.01
20.11.05	389	68	2546	16.3	31.0	26.8	68.9	0.00	41.18	30.43	10.71	6.07	727.38	353.50	165.44	6.07	0.25	686.20	0.04	323.08	0.02	154.73	0.01

21.02.18	307	63	934	-1.0	64.2	27.2	81.1	0.00	62.55	32.50	9.75	5.79	400.60	252.51	70.28	5.79	0.12	338.05	0.01	220.01	0.01	60.53	0.00
21.03.11	374	28	2030	15.9	39.3	28.7	67.3	0.02	109.01	73.13	43.38	0.59	467.59	284.03	218.29	0.57	0.02	358.58	0.02	210.90	0.01	174.91	0.01
21.03.30	374	47	2935	18.1	33.1	28.1	76.3	0.00	358.84	244.88	41.88	5.07	974.07	733.80	164.70	5.07	0.25	615.24	0.04	488.92	0.03	122.82	0.01
21.04.14	374	62	2876	14.8	35.8	28.0	69.3	0.00	199.51	146.30	91.67	6.63	469.87	480.77	167.20	6.63	0.32	270.37	0.02	334.47	0.02	75.53	0.01
21.05.13	389	35	14109	29.0	46.0	30.6	67.8	0.00	59.91	40.00	20.00	1.89	424.77	240.62	93.40	1.89	0.41	364.86	0.12	200.62	0.06	73.40	0.02
21.06.09	351	55	17604	31.1	52.6	31.6	56.4	0.00	67.57	33.17	19.50	0.39	327.31	195.95	106.83	0.39	0.12	259.74	0.11	162.78	0.07	87.33	0.04
21.07.29	351	63	24540	33.8	52.0	33.5	63.0	0.00	60.19	30.00	16.33	0.61	n/a	266.22	74.44	0.61	0.25	n/a	n/a	236.22	0.14	58.11	0.04
21.09.15	353	62	6576	30.1	55.1	29.6	55.1	0.00	48.44	19.14	5.86	0.79	498.12	187.56	107.51	0.79	0.09	449.69	0.07	168.42	0.03	101.65	0.02
21.09.30	353	77	5545	27.3	57.4	28.8	63.7	1.36	52.85	19.43	11.29	2.67	446.22	202.56	86.11	1.30	0.12	393.38	0.05	183.13	0.03	74.83	0.01
21.10.19	376	46	1154	16.4	62.2	28.7	81.2	0.00	55.75	26.38	14.25	6.30	345.56	243.22	106.56	6.30	0.12	289.80	0.01	216.85	0.01	92.31	0.00

Table S2. All measurement data obtained from growing-finishing pigs. "n/a" indicates cases where there are no measurement results or they were lost due to errors in the measurement process. (NA: number of animals, AA: age of animals in days, VR: ventilation rate in m^3 h^{-1}, OT: outdoor air temperature in °C, OH: outdoor relative humidity in %, IT: indoor air temperature in °C, IH: indoor relative humidity in %, OC: outdoor concentration in ppm for ammonia and μg m^{-3} for PMs, EXC: concentration at air outlets in ppm for ammonia and μg m^{-3} for PMs, EC: emission concentration in ppm for ammonia and μg m^{-3} for PMs, and EF: emission factor in kg $animal^{-1}$ yr^{-1}).

Date (yy.mm.dd)	NA	AA	VR	OT	OH	IT	IH	OC				EXC				NH_3		TSP		PM_{10}		$PM_{2.5}$	
								NH_3	TSP	PM_{10}	$PM_{2.5}$	NH_3	TSP	PM_{10}	$PM_{2.5}$	EC	EF	EC	EF	EC	EF	EC	EF
20.05.21	1059	153	91112	24	54	29	42	0.00	35.03	20.63	10.88	8.64	403.29	n/a	103.60	8.64	4.52	368.26	0.28	n/a	n/a	92.73	0.07
20.06.05	1059	168	196555	29	57	31	48	0.36	196.22	127.00	27.60	1.08	676.65	209.20	n/a	0.72	0.80	480.43	0.78	82.20	0.13	n/a	n/a
20.06.16	1064	177	129717	27	53	29	62	0.12	80.02	37.63	18.38	1.53	229.94	139.33	n/a	1.41	1.03	149.91	0.16	101.70	0.11	n/a	n/a
20.06.23	1040	151	178445	31	50	30	52	0.22	102.02	30.13	16.63	0.88	n/a	120.81	291.34	0.66	0.67	n/a	n/a	90.69	0.14	274.71	0.41
20.08.20	1085	135	289327	35	57	32	63	0.15	58.22	23.38	16.00	0.33	67.11	52.85	n/a	0.18	0.28	8.89	0.02	29.48	0.07	n/a	n/a
20.08.28	1085	143	218592	33	67	32	72	0.00	35.96	17.91	7.73	0.51	70.04	57.70	40.08	0.51	0.61	34.08	0.06	39.79	0.07	32.36	0.06
20.09.10	1065	134	95282	26	70	29	66	0.00	71.97	27.14	13.57	2.01	129.57	74.46	53.76	2.01	1.08	57.60	0.05	47.32	0.04	40.19	0.03
20.10.22	1169	134	42410	18	65	26	67	0.00	188.17	124.00	34.86	4.15	344.44	243.56	115.00	4.15	0.93	156.27	0.05	119.56	0.04	80.14	0.03
20.11.05	1271	137	42286	16	31	25	55	0.00	41.18	30.43	10.71	6.49	414.23	294.22	134.45	6.49	1.34	373.05	0.11	263.79	0.08	123.73	0.04
21.02.18	1340	158	20827	-1	64	26	64	0.00	62.55	32.50	9.75	19.22	1876.91	1164.37	393.09	19.22	1.98	1814.36	0.25	1131.87	0.15	383.34	0.05
21.03.11	1306	124	39024	16	39	26	48	0.02	109.01	73.13	43.38	9.78	980.41	593.75	344.37	9.77	1.82	871.40	0.23	520.62	0.14	301.00	0.08

21.03.30	1206	144	44950	18	33	26	42	0.00	358.84	244.88	41.88	6.54	769.68	535.65	168.98	6.54	1.51	410.84	0.13	290.77	0.09	127.11	0.04
21.04.14	1236	129	38960	15	36	26	50	0.00	199.51	146.30	91.67	20.54	1489.12	758.91	190.49	20.54	4.05	1289.61	0.36	612.62	0.17	98.82	0.03
21.06.09	1182	139	208269	31	53	31	62	0.00	67.57	33.17	19.50	1.15	319.44	129.75	78.12	1.15	1.20	251.87	0.39	96.58	0.15	58.62	0.09
21.07.21	1182	188	226800	34	46	34	57	0.00	47.52	20.78	14.83	1.58	n/a	178.01	103.94	1.58	1.78	n/a	n/a	157.23	0.26	89.10	0.15
21.07.29	1182	139	300608	34	52	33	62	0.00	60.19	30.00	16.33	1.18	n/a	380.48	129.68	1.18	1.76	n/a	n/a	350.48	0.78	113.35	0.25
21.09.15	1127	133	116986	30	55	30	61	0.00	48.44	19.14	5.86	6.33	467.11	170.67	96.00	6.33	3.91	418.68	0.38	151.52	0.14	90.14	0.08
21.09.30	1127	148	71013	27	57	29	63	1.36	52.85	19.43	11.29	8.32	307.52	n/a	112.15	6.96	2.63	254.68	0.14	n/a	n/a	100.87	0.06
21.10.19	1182	166	36689	16	62	27	57	0.00	62.55	26.38	14.25	17.18	415.74	382.41	153.01	17.18	3.32	353.19	0.10	356.03	0.10	138.76	0.04

Table S3. Monthly marketed-pigs per sow per year (MSY) of the experimental pig farm.

Months	MSY
May-2020	24.9
Jun-2020	16.9
Jul-2020	32.0
Aug-2020	16.8
Sep-2020	19.8
Oct-2020	29.3
Nov-2020	28.2
Dec-2020	27.3
Jan-2021	18.0
Feb-2021	23.6
Mar-2021	26.1
Apr-2021	36.3
May-2021	31.8
Jun-2021	19.6
Jul-2021	32.3
Aug-2021	19.8
Sep-2021	22.0
Oct-2021	25.7
Nov-2021	24.6
